# A novel risk model based on white blood cell-related biomarkers for acute kidney injury prediction in patients with ischemic stroke admitted to the intensive care unit

**DOI:** 10.3389/fmed.2022.1043396

**Published:** 2022-12-12

**Authors:** Shengyuan Liu, Min Li, Yuxing Yang, Yiguo Chen, Wei Wang, Xiaoyu Zheng

**Affiliations:** ^1^Department of Neurosurgery, Affiliated Hospital of Zunyi Medical University, Zunyi, Guizhou, China; ^2^Department of Physiology, Zunyi Medical and Pharmaceutical College, Zunyi, Guizhou, China; ^3^Department of Urology, The People’s Hospital of Yubei District of Chongqing City, Chongqing, China; ^4^Department of Orthopedics, The People’s Hospital of Yubei District of Chongqing City, Chongqing, China; ^5^School of Clinical Medicine, Chongqing Medical and Pharmaceutical College, Chongqing, China

**Keywords:** white blood cell, ischemic stroke, risk model, acute kidney injury, intensive care unit

## Abstract

**Background:**

Conventional systemic inflammatory biomarkers could predict prognosis in patients with ischemic stroke (IS) admitted to the intensive care unit (ICU). Acute kidney injury (AKI) is common in patients with IS admitted to ICU, but few studies have used systemic inflammatory biomarkers to predict AKI in critically ill patients with IS. This study aimed to establish a risk model based on white blood cell (WBC)-related biomarkers to predict AKI in critically ill patients with IS.

**Methods:**

Data were extracted from the Medical Information Mart for Intensive Care-IV (MIMIC-IV) for a training cohort, and data were extracted from the Medical Information Mart for eICU Collaborative Research Database (eICU-CRD) for a validation cohort. Logistic regression analysis was used to determine the significant predictors of WBC-related biomarkers on AKI prediction, and a risk model was established based on those significant indicators in multivariate logistic regression. The receiver operating characteristics (ROC) curve was utilized to obtain the best cut-off value of the risk model. The Kaplan–Meier curve was used to evaluate the prognosis-predictive ability of the risk model.

**Results:**

The overall incidence of AKI was 28.4% in the training cohort and 33.2% in the validation cohort. WBC to lymphocyte ratio (WLR), WBC to basophils ratio (WBR), WBC to hemoglobin ratio (WHR), and neutrophil to lymphocyte ratio (NLR) could independently predict AKI, and a novel risk model was established based on WLR, WBR, WHR, and NLR. This risk model depicted good prediction performance both in AKI and other clinical outcomes including hemorrhage, persistent AKI, AKI progression, ICU mortality, and in-hospital mortality both in the training set and in the validation set.

**Conclusion:**

A risk model based on WBC-related indicators exhibited good AKI prediction performance in critically ill patients with IS which could provide a risk stratification tool for clinicians in the ICU.

## Introduction

Ischemic stroke (IS) is a leading cause of mortality and morbidity, with long-term debilitating effects, which caused huge social and economic costs (1). Acute kidney injury (AKI) is defined by an increase in serum creatinine and a decrease in urine output (2). The epidemiology of AKI following IS is commonly overlooked following stroke events. The incidence of AKI is a common complication among patients with IS, which is tightly linked to poor prognosis (3). A previous review including eight studies reported that the incidence of AKI in the IS setting was 9.62%, and mortality in patients with IS who develop AKI increased 2.5-fold more than in non-AKI (4). The incidence of AKI and mortality in patients with IS admitted to the ICU would be higher. Wang et al. retrospectively recruited 647 patients with stroke from the neurology ICU, 20.9% of critically ill patients with IS developed AKI, and patients in the neurology ICU who developed AKI increased 4.9-folds, 19.7-folds, and 48.6-folds all-cause mortality at AKI-stage 1, AKI-stage 2, and AKI-stage 3, respectively (5). Hence, it is important to find novel biomarkers that can predict AKI to improve post-IS clinical outcomes and reduce mortality rates in these critically ill patients with IS.

A previous study reported that urinary liver-type fatty-acid binding protein (L-FABP) was independently associated with the development of AKI and 90-day mortality in critically ill patients with IS (6). Xiao et al. found that serum neutrophil gelatinase-associated lipocalin (NGAL) could predict AKI in patients with IS (7). However, the above-mentioned biomarkers are not easy to obtain in the ICU setting, and finding relatively clinically often-used indicators may help ICU clinicians in identifying those at high risk. Inflammation and immune responses have been found to be involved in the pathological progression of IS in the ICU setting (8, 9). Under IS conditions, the dynamic balance between the pro-inflammatory and anti-inflammatory responses was disturbed, and inhibited inflammation can alleviate brain injury and improve prognosis (10). Numerous studies reported that blood routine-based inflammatory indicators such as neutrophil to lymphocyte ratio (NLR), platelet to lymphocyte ratio (PLR), and lymphocyte to monocyte ratio (LMR) could predict the prognosis of patients with IS (11–13). However, whether these white blood cell (WBC)-related indicators could predict AKI in the setting of AIS admitted to ICU remains largely unknown.

Hence, this study aims to evaluate the AKI predictive ability of the WBC-related indicators in critically ill patients with IS and then develop a risk model based on those significant WBC-related indicators to facilitate ICU clinicians for better clinical decisions.

## Materials and methods

### Data source

The data used in this study were obtained from the Medical Information Mart for Intensive Care (MIMIC)-IV database (version: 1.0) from 2008 to 2019 (14) and the eICU Collaborative Research Database (eICU-CRD) (15). eICU-CRD contains data on more than 200 thousand ICU admissions in 2014 and 2015 at 208 US hospitals, while MIMIC-IV includes information on more than 70,000 patients admitted to the ICUs of Beth Israel Deaconess Medical Center in Boston, MA, from 2008 to 2019. The MIMIC-IV database and eICU-CRD database are accessible to individuals who have completed the Collaborative Institutional Training Initiative examination. The study was conducted in accordance with the Declaration of Helsinki. We obtained permission to extract data from the MIMIC-IV database and eICU-CRD database.

**FIGURE 1:**
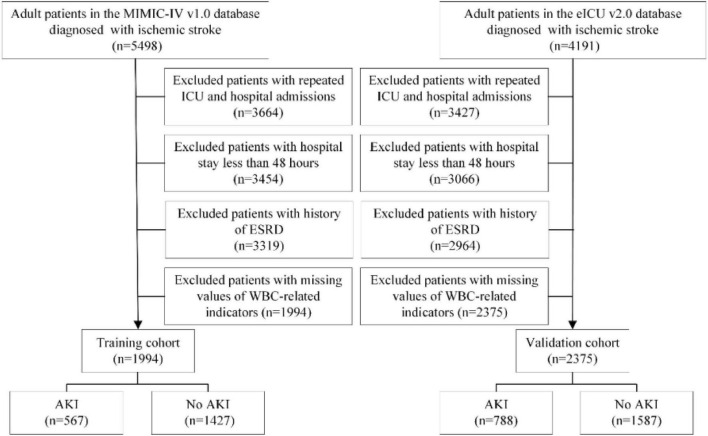
The flow chart of this study.

The inclusion criteria were as follows: Adult patients with IS, defined as ICD-9 codes of 433, 434, 436, 437.0, and 437.1, or ICD-10 codes of I63, I65, and I66. AKI diagnosis was based on KDIGO-AKI criteria according to serum creatinine in the first 48 h of their ICU admission (16). Patients with one of the following conditions were excluded: (1) Less than 18 years at first admission to ICU; (2) less than 48 h of hospital stay; (3) patients with repeated ICU or hospital admissions; (4) patients with end-stage renal disease (ESRD); and (5) patients with missing values of WBC-related indicators. We selected the MIMIC-IV database as the training cohort and the eICU-CRD database as the validation cohort. The detailed flowchart is shown in Figure 1.

### Data collection and outcomes

The following information was collected: General information, vital signs, scoring systems [the sequential organ failure assessment (SOFA) score, oxford acute severity of illness score (OASIS), acute physiology score III (APS III) score, and simplified acute physiology score II (SAPS II)], systemic inflammatory response syndrome (SIRS) score, HAS-BLED score, comorbidities, laboratory data, and drugs usage. All of the laboratory results extracted were from the first test results after the patient entered the ICU. The primary outcome was AKI. The secondary outcomes were AKI severity, AKI progression, intracerebral hemorrhage, in-hospital mortality, and ICU mortality.

### Definition

Systemic immune-inflammation index (SII) was calculated as platelet counts* neutrophil count/lymphocyte counts. According to previous reports, AKI progression, AKI recovery, and persistent AKI (pAKI) were diagnosed, in brief, AKI progression was defined as a worsening of AKI-stage: From stage 1 to stage 2 or 3 or from stage 2 to 3. Patients diagnosed with progressive or persisting stage 3 AKI (stage 3 AKI for ≥2 consecutive days) were classified as having AKI progression. AKI recovery was defined as greater than or equal to a 50% decrease in serum creatinine after the diagnosis of AKI and/or return of serum creatinine to the baseline value. Persistent AKI was defined as renal dysfunction without recovery within 2 days or before death (17, 18). Moreover, intracerebral hemorrhage, acute heart failure, sepsis, and acute respiratory failure were defined as the ICD-9/10 codes.

### Statistical analysis

Variables included in this study were represented as *n* (%) or mean ± standard deviation (SD). A comparison of two groups using the Kruskal–Wallis test or the Chi-square test. *P* < 0.05 was considered to indicate a statistically significant difference on both sides.

A novel AKI prediction risk model [risk = 0.884 × Lg (WLR) + 0.027 × WBR + 1.460 × WHR + 0.013 × NLR] was obtained from the results of a multivariate logistic regression analysis. The decision curve analysis (DCA) was performed to determine the clinical utilities of the risk model. Moreover, univariate and multivariate logistic or Cox regressions were conducted to determine the risk model for the prediction of different clinical outcomes. Subgroup analysis was exhibited by the forest plot. Waterfall plots were used to display the distribution of clinical outcomes and their corresponding risk scores (19).

## Results

### Baseline characteristics

A total of 1,994 critically ill patients with IS from the MIMIC-IV database and 2,375 critically ill patients with IS from the eICU-CRD database were enrolled in this study, with 567 (28.4%) developed AKI in the training set and 788 (33.2%) developed AKI in the validation set. Compared to those who developed non-AKI, patients who developed AKI had a significantly higher prevalence of congestive heart failure, diabetes, chronic kidney disease, a higher proportion of diuretic use, increased weight, SOFA, OASIS, APS III, HAS-BLED scores, lower MAP, and higher heart rate in both the training and validation sets (*P* < 0.05 for all comparisons, Table 1). Moreover, patients who developed AKI had markedly higher WBC counts, BUN, creatinine, and potassium, and lower hemoglobin, platelet, albumin, and bilirubin compared to the non-AKI group in both training and validation sets (*P* < 0.05 for all comparisons, Table 1).

**TABLE 1:** Comparisons of characteristics between the non-AKI group and AKI group.

Characteristics	Training set (*n* = 1,994)			Validation set (*n* = 2,375)		
		
	Non-AKI (*n* = 1,427)	AKI (*n* = 567)	*P*-value	Non-AKI (*n* = 1,587)	AKI (*n* = 788)	*P*-value
Age, years old	69.5 ± 15.5	68.1 ± 15.1	0.077	68.9 ± 14.3	69.7 ± 13.7	0.194
Gender, male, *n* (%)	700 (49.1)	327 (57.7)	0.001	782 (49.3)	362 (45.9)	0.137
Weight, kg	78.5 ± 21.8	81.9 ± 23.7	0.002	80.3 ± 21.8	85.7 ± 23.7	<0.001
Ethnicity, *n* (%)			0.452			0.329
White	851 (59.6)	353 (62.3)		1,294 (81.5)	652 (82.7)	
Black	238 (16.7)	94 (16.6)		164 (10.3)	84 (10.7)	
Others	338 (23.7)	120 (21.2)		129 (8.1)	52 (6.6)	
**Comorbidities, *n* (%)**						
Myocardial infarction	248 (17.4)	160 (28.2)	<0.001	164 (10.3)	66 (8.4)	0.148
Congestive heart failure	376 (26.3)	244 (43.0)	<0.001	168 (10.6)	111 (14.1)	0.015
Hypertension	616 (43.2)	314 (55.4)	<0.001	1,008 (63.5)	501 (63.6)	1.000
Diabetes	486 (34.1)	253 (44.6)	<0.001	416 (26.2)	247 (31.3)	0.013
Chronic kidney disease	229 (16.0)	236 (41.6)	<0.001	114 (7.2)	84 (10.7)	0.005
Atrial fibrillation	573 (40.2)	208 (36.7)	0.167	284 (17.9)	158 (20.1)	0.224
Thrombosis	297 (20.8)	121 (21.3)	0.841	41 (2.6)	22 (2.8)	0.871
CCI, points	7.2 ± 2.8	8.1 ± 2.9	<0.001	4.0 ± 1.4	4.4 ± 1.5	<0.001
**Drugs usage, *n* (%)**						
Diuretic	717 (50.2)	429 (75.7)	<0.001	358 (22.6)	259 (32.9)	<0.001
Antiplatelet	1,044 (73.2)	419 (73.9)	0.780	264 (16.6)	182 (23.1)	<0.001
Anticoagulation	508 (35.6)	188 (33.2)	0.327	199 (12.5)	103 (13.1)	0.764
**Score system, points**						
SOFA	4.3 ± 1.1	8.4 ± 2.1	<0.001	2.4 ± 0.7	4.3 ± 1.1	<0.001
OASIS	32.7 ± 8.5	38.6 ± 10.3	<0.001	22.4 ± 8.8	28.3 ± 10.5	<0.001
APSIII	46.0 ± 11.2	69.7 ± 27.8	<0.001	26.2 ± 10.4	48.3 ± 14.2	<0.001
SAPSII	34.1 ± 11.5	45.6 ± 14.8	<0.001	–	–	−
HAS-BLED score	3.6 ± 1.0	3.7 ± 1.0	0.005	2.7 ± 1.0	2.9 ± 1.1	<0.001
**Vital signs**						
MAP, mmHg	91.0 ± 18.4	84.6 ± 20.0	<0.001	104.4 ± 19.7	101.2 ± 21.9	0.017
Heart rate, bpm	85.3 ± 19.3	90.2 ± 20.9	<0.001	82.7 ± 18.5	82.3 ± 21.0	<0.001
RR, bpm	19.5 ± 5.6	20.7 ± 7.0	<0.001	19.2 ± 5.1	19.5 ± 5.3	0.153
SpO_2_%	97.3 ± 3.4	96.8 ± 4.2	0.004	97.0 ± 4.4	97.0 ± 3.3	0.856
**Laboratory values**						
White blood cell, ×10^9^/L	11.7 ± 3.9	12.5 ± 4.5	<0.001	10.1 ± 2.9	11.3 ± 3.6	<0.001
Hemoglobin, g/dL	11.7 ± 2.3	10.7 ± 2.4	<0.001	13.2 ± 2.2	12.9 ± 2.5	0.010
Platelet, ×10^9^/L	223.6 ± 77.8	202.7 ± 73.1	<0.001	231.9 ± 72.7	221.3 ± 77.9	0.004
Albumin, g/dL	3.4 ± 0.5	3.0 ± 0.7	<0.001	3.5 ± 0.5	3.4 ± 0.6	<0.001
Bilirubin, mmol/L	0.9 ± 0.3	0.7 ± 0.2	0.024	0.7 ± 0.2	0.8 ± 0.3	0.002
BUN, mg/dL	22.4 ± 6.8	32.7 ± 9.8	<0.001	21.0 ± 6.5	23.6 ± 6.2	<0.001
Creatinine, mg/dL	1.1 ± 0.3	2.2 ± 0.8	<0.001	1.1 ± 0.3	1.3 ± 0.4	<0.001
Potassium, mmol/L	4.2 ± 0.7	4.3 ± 0.9	<0.001	4.0 ± 0.6	4.1 ± 0.6	0.006
Sodium, mmol/L	139.4 ± 5.1	138.5 ± 5.4	<0.001	138.4 ± 4.3	138.4 ± 4.5	0.856

AKI, acute kidney injury; CCI, Charlson comorbidity index; SOFA, sequential organ failure assessment; OASIS, oxford acute severity of illness score; APSIII, acute physiology score III; SAPSII, simplified acute physiology score II; SIRS, systemic inflammatory response syndrome; MAP, mean arterial pressure; RR, respiratory rate; SpO2, saturation of peripheral oxygen; BUN, blood urea nitrogen.

### A novel risk model was established based on WBC-related indicators

Multivariate logistic regression was conducted to determine the WBC-related indicators for AKI prediction. As shown in Table 2, Lg (WLR), WBR, WHR, and NLR were independent prognostic predictors of AKI, and a novel risk model was established based on the significant factors of multivariate logistic regression: Risk score = 0.884 × Lg (WLR) + 0.027 × WBR + 1.460 × WHR + 0.013 × NLR.

**TABLE 2:** Logistic regression analysis of WBC-related indicators for AKI.

	Univariate			Multivariate		
		
	β	OR (95% CI)	*P*-value	β	OR (95% CI)	*P*-value
WNR	0.009	1.01 (0.99–1.03)	0.460			
WMR	0.000	1.00 (1.00–1.00)	0.633			
Lg (WMR)	0.587	1.80 (1.39–2.33)	<0.001	0.299	1.35 (0.89–2.03)	0.154
WLR	0.001	1.00 (1.00–1.00)	0.076			
Lg (WLR)	0.804	2.24 (1.72–2.91)	<0.001	0.884	2.42 (1.44–4.06)	<0.001
WER	0.000	1.00 (1.00–1.00)	0.196			
Lg (WER)	0.353	1.42 (1.23–1.64)	<0.001	0.289	1.23 (0.48–1.92)	0.243
WBR	0.021	1.02 (1.01–1.03)	<0.001	0.027	1.03 (3.21–1.03)	<0.001
WHR	1.571	4.81 (3.65–6.34)	<0.001	1.460	4.31 (3.21–5.78)	<0.001
NLR	0.033	1.03 (1.02–1.05)	<0.001	0.013	1.02 (1.01–1.03)	0.033
PLR	0.000	1.00 (1.00–1.00)	0.229			
Lg (PLR)	0.289	1.34 (1.04–1.72)	0.024	–0.194	0.82 (0.48–1.42)	0.484
MLR	0.030	1.03 (0.99–1.07)	0.105			
SII	0.000	1.00 (1.00–1.00)	0.133			
Lg (SII)	0.339	1.40 (1.15–1.71)	0.001	–0.202	0.82 (0.52–1.29)	0.387

WBC, white blood count; AKI, acute kidney injury; OR, odds ratio; 95% CI, 95% confidence index; WNR, WBC to neutrophil ratio; WMR, WBC to monocyte ratio; WLR, WBC to lymphocyte ratio; WER, WBC to eosinophils ratio; WBR, WBC to basophils ratio; WHR, WBC to hemoglobin ratio; NLR, neutrophil to lymphocyte ratio; PLR, platelets to lymphocyte ratio; MLR, monocyte to lymphocyte ratio; SII, systemic immune-inflammation index.

**FIGURE 2:**
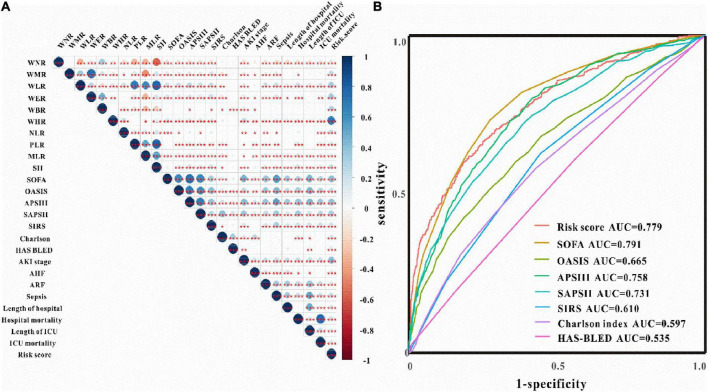
Clinical correlation and predictive ability of WBC-related biomarkers. **(A)** The correlations between risk score with WBC-related biomarkers, severity score, and clinical outcomes. **(B)** ROC curves of risk score and severity score for the prediction of AKI among critically ill patients with IS.

Verification of the new model’s performance in AKI prediction We first checked the correlations between risk scores with WBC-related biomarkers, severity scores, and clinical outcomes. As shown in Figure 2A, the risk model is tightly related to WBC-related biomarkers, severity scores, and clinical outcomes. ROC curves evaluated the AKI prediction abilities of the risk model and other score systems among critically ill patients with IS. The results indicated that the WBC-related biomarkers-based risk model exhibited better AKI prediction performance (AUC: 0.779, sensitivity: 62.1%, specificity: 79.8%) than other score systems including OASIS score, APS III score, SAPS II score, SIRS score, Charlson index, and HAS-BLED score (Figure 2B). The best cut-off value of the novel risk model is 2.65 according to the ROC analysis, and the low-risk (≤2.65) and high-risk (>2.65) groups were divided according to the best cut-off value. As shown in Table 3, the serum creatinine levels were significantly higher in the high-risk group compared to the low-risk group regardless of before or after the diagnosis of AKI compared to those in the low-risk group (*P* < 0.05). Critically ill patients with IS at high risk developed AKI, persistent AKI, AKI progression, intracerebral hemorrhage, had higher stage AKI, in-hospital mortality, and ICU mortality both in the training and validation sets (*P* < 0.05 for all, Table 4 and Supplementary Table 1).

**TABLE 3:** Change in serum creatinine before and after the diagnosis of AKI for critically ill patients with IS.

Variables	High-risk group	Low-risk group	*P*-value
Serum creatinine at ICU admission, mean ± SD	2.82 ± 1.05	1.10 ± 0.44	<0.001
Serum creatinine at first AKI diagnosis, mean ± SD	3.54 ± 2.77	1.74 ± 1.00	<0.001
Serum creatinine at 48 h after AKI diagnosis, mean ± SD	2.75 ± 1.87	1.73 ± 1.12	<0.001
Positive changes of serum creatinine, *n* (%)	170 (48.3)	62 (28.8)	
Δserum creatinine, mean ± SD	1.95 ± 5.84	0.49 ± 0.43	<0.001
Negative or static change of serum creatinine, *n* (%)	182 (51.7)	153 (71.2)	
Δserum creatinine, mean ± SD	−0.29 ± 0.54	−0.18 ± 0.38	<0.001

ICU, intensive care unit; SD, standard deviation; IQR, interquartile range; AKI, acute kidney injury.

**TABLE 4:** Clinical outcomes analysis of high- and low-risk groups for all patients.

Outcomes	Low-risk group	High-risk group	Effect size	*P*-value
N	1,353	641	–	−
**Primary outcome**				
AKI	215 (15.9)	352 (54.9)	0.894	<0.001
**Secondary outcomes**				
AKI severity^1^			0.688	<0.001
Stage I	171 (79.5)	170 (48.3)		
Stage II	24 (11.2)	99 (28.1)		
Stage III	20 (9.3)	83 (23.6)		
Persistent AKI^1^	70 (32.6)	185 (52.6)	0.413	<0.001
AKI progression^1^	45 (20.9)	120 (34.1)	0.287	<0.001
Intracerebral hemorrhage	228 (16.9)	149 (23.2)	0.160	0.001
In-hospital mortality	201 (14.9)	172 (26.8)	0.298	<0.001
ICU mortality	132 (9.8)	102 (15.9)	0.185	<0.001

AKI, acute kidney injury; ICU, intensive care unit. ^1^Excluded patients without the incidence of AKI.

**FIGURE 3:**
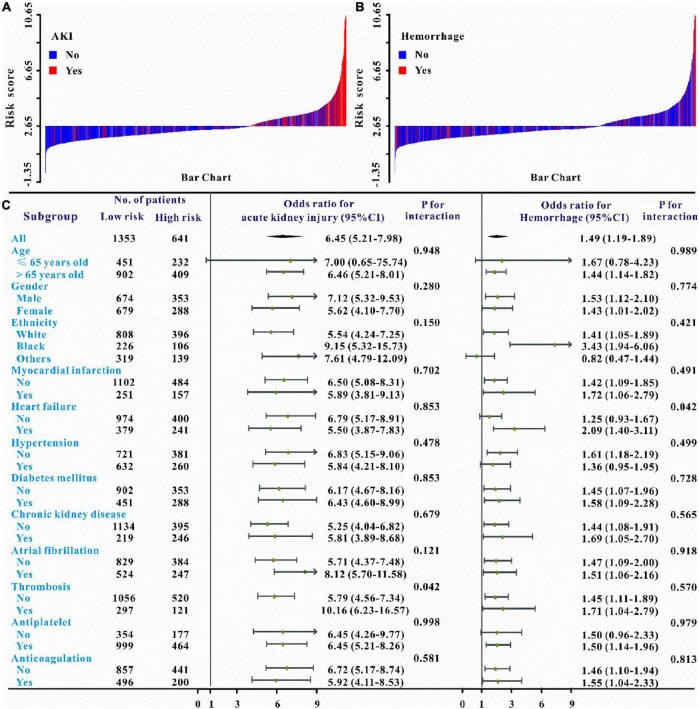
Waterfall plots and forest plots of the high-risk group and low-risk group for the prediction of AKI and intracerebral hemorrhage among critically ill patients with IS. The waterfall plot of the risk score for each patient of AKI **(A)** and intracerebral hemorrhage **(B)**. The subgroup analysis of the risk score in individuals with critically ill IS for AKI and intracerebral hemorrhage **(C)**.

We then utilized univariate and multivariate logistic regression analyses to assess the AKI predictive ability of the risk model. The results indicated that when adjusted for different models, the risk model could remain to predict AKI in both training and validation sets (*P* < 0.05 for all models, Table 5 and Supplementary Tables 2, 3). Furthermore, the waterfall plots were used to reflect the relationship between the risk score and the prevalence of the developed-AKI proportion. As shown in Figure 3A, a great majority of patients who developed AKI had higher risk scores than those who developed non-AKI. The risk model could independently predict AKI in critically ill patients with IS (OR: 6.45, 95% CI: 5.21–7.98; *P* < 0.05, Figure 3C). The novel risk model can predict AKI in almost all subgroups except for patients younger than 65 years of age (all *P* < 0.01, Figure 3C). These results indicated that the WBC-related biomarkers-based risk model could significantly predict AKI in critically ill patients with IS.

**TABLE 5:** Univariate and multivariate logistic regression analyses for clinical outcomes.

Methods	OR (95% CI)	*P*-value
**For AKI**		
Unadjusted	6.45 (5.21–7.98)	<0.001
Adjusted for model I	6.36 (5.14–7.88)	<0.001
Adjusted for model II	3.42 (2.64–4.42)	<0.001
Adjusted for model III	3.62 (2.76–4.75)	<0.001
**For intracerebral hemorrhage**		
Unadjusted	1.49 (1.19–1.89)	0.001
Adjusted for model I	1.47 (1.16–1.85)	0.001
Adjusted for model II	1.35 (1.04–1.76)	0.025
Adjusted for model III	1.40 (1.07–1.84)	0.015
**For persistent AKI** ^1^		
Unadjusted	2.30 (1.61–3.27)	<0.001
Adjusted for model I	2.22 (1.55–3.18)	<0.001
Adjusted for model II	2.57 (1.70–3.88)	<0.001
Adjusted for model III	2.40 (1.54–3.73)	<0.001
**For AKI progression** ^1^		
Unadjusted	2.66 (1.74–4.06)	<0.001
Adjusted for model I	2.64 (1.72–4.04)	<0.001
Adjusted for model II	1.81 (1.13–2.92)	0.014
Adjusted for model III	1.85 (1.12–3.04)	0.016

AKI, acute kidney injury; OR, odds ratio; 95%CI, 95% confidence index. Model I adjusted for age, gender, weight, and ethnicity. Model II adjusted for the model I plus comorbidities and Charlson comorbidity index, HAS-BLED score, score system, interventions, and drug usage. Model III adjusted for model II plus vital signs and laboratory results except for white blood count.

^1^Excluded patients without the incidence of AKI.

**FIGURE 4:**
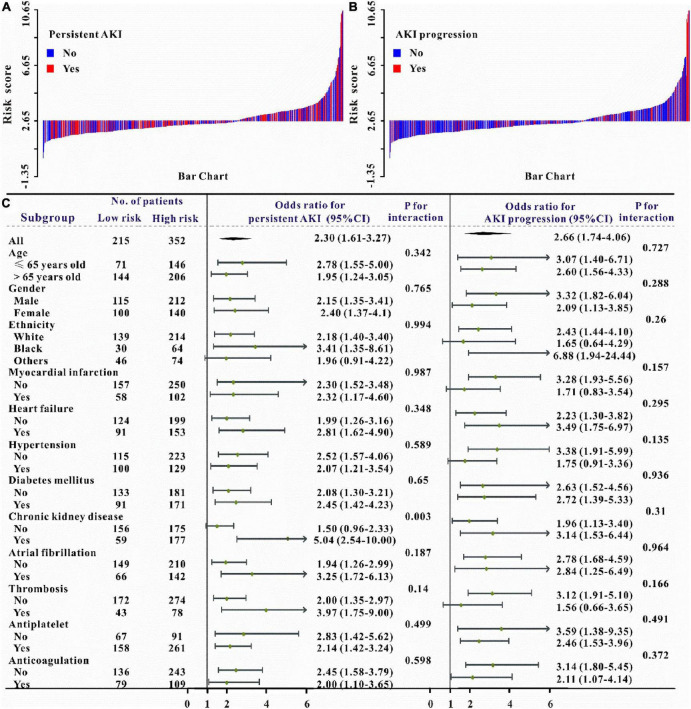
Waterfall plots and forest plots of the high-risk group and low-risk group for the prediction of persistent acute kidney injury (pAKI) and AKI progression. The waterfall plot of the risk score for each patient of pAKI **(A)** and AKI progression **(B)**. The subgroup analysis of the risk score in individuals with AKI for pAKI and AKI progression **(C)**.

Verification of the new model’s performance in other clinical outcomes prediction. We also intended to evaluate the predictive abilities of this risk model on the secondary clinical outcomes including hemorrhage, persistent AKI, AKI progression, ICU mortality, and in-hospital mortality. Multivariate logistic or Cox regression analysis revealed that this risk model could also independently predict all secondary clinical outcomes and when adjusted for different models, this result remains solid (Tables 5–6 and Supplementary Table 2). The waterfall plot results verified that higher risk scores were associated with a higher incidence of hemorrhage, persistent AKI, and AKI progression in both training and validation sets (Figures 3B, 4A–B and Supplementary Figure 2). The predictive abilities of the new risk model were further verified across different subgroups: Hemorrhage (OR: 1.49, 95% CI: 1.19–1.89; *P* < 0.05, Figure 3C), persistent AKI (OR: 2.30, 95% CI: 1.61–3.27; *P* < 0.05, Figure 4C), and AKI progression (OR: 2.66, 95% CI: 1.74–4.06; *P* < 0.05, Figure 4C).

**TABLE 6:** Univariate and multivariate Cox regression analyses for clinical outcomes.

Methods	HR (95% CI)	*P*-value
**For ICU mortality**		
Unadjusted	1.96 (1.51–2.55)	<0.001
Adjusted for model I	1.68 (1.16–2.43)	0.006
Adjusted for model II	2.18 (1.42–3.34)	<0.001
Adjusted for model III	2.25 (1.41–3.60)	0.001
**For in-hospital mortality**		
Unadjusted	2.10 (1.71–2.58)	<0.001
Adjusted for model I	2.03 (1.54–3.02)	<0.001
Adjusted for model II	1.55 (1.12–2.13)	0.008
Adjusted for model III	1.60 (1.15–2.24)	0.005

AKI, acute kidney injury; HR, hazard ratio; 95% CI, 95% confidence index. Model I adjusted for age, gender, weight, and ethnicity. Model II adjusted for the model I plus comorbidities and Charlson comorbidity index, HAS-BLED score, score system, interventions, and drug usage. Model III adjusted for model II plus vital signs and laboratory results except for white blood count.

**FIGURE 5:**
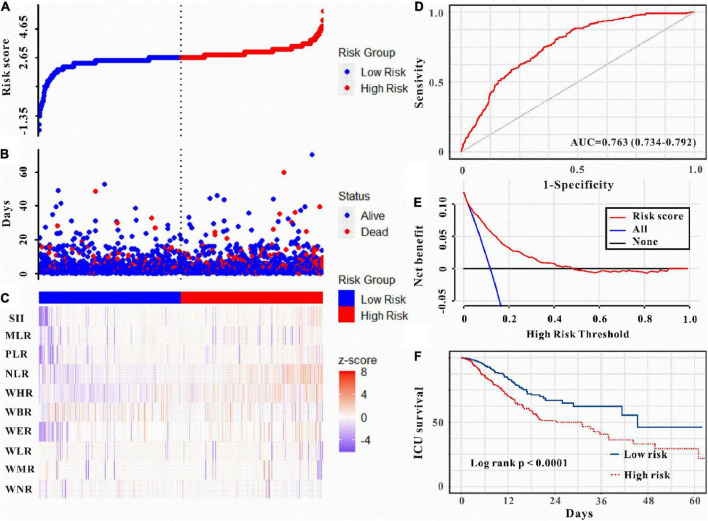
Risk score was established to detect the ICU mortality of critically ill patients with IS. All patients were distinguished into high and low risk based on the risk score **(A)**, the relationship between survival time and prognosis of patients in the two corresponding groups **(B)**, and the heatmap of WBC-related indicators between the two groups **(C)**. Receiver operating characteristic (ROC) curve analysis of the risk score for ICU mortality **(D)**, decision curve analysis of the risk score for ICU mortality **(E)**. Kaplan–Meier curves show the ICU mortality of groups with different risks **(F)**.

**FIGURE 6:**
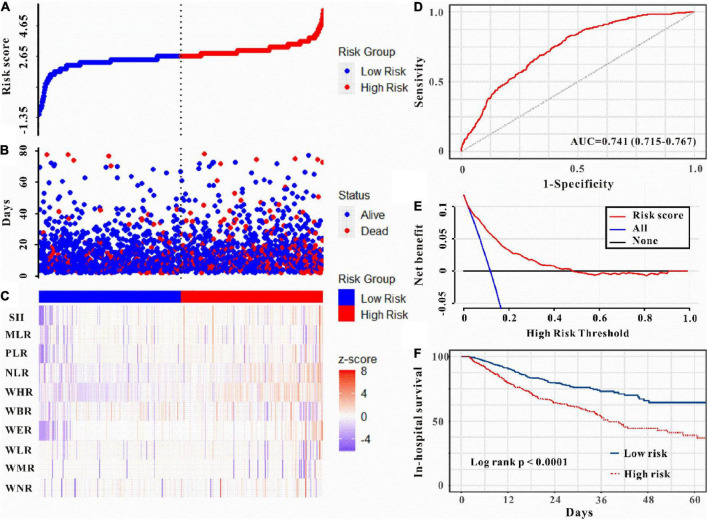
Risk score was established to detect in-hospital mortality in critically ill patients with IS. All patients were distinguished into high and low risk based on the risk score **(A)**, the relationship between survival time and prognosis of patients in the two corresponding groups **(B)**, and the heatmap of inflammatory marks between the two groups **(C)**. Receiver operating characteristic (ROC) curve analysis of the risk score for in-hospital mortality **(D)** and decision curve analysis of the risk score for in-hospital mortality **(E)**. Kaplan–Meier curves show the in-hospital mortality of groups with different risks **(F)**.

Moreover, as can be seen in Figures 5–6, the risk model depicted good ICU mortality (AUC: 0.763, 95% CI: 0.734–0.792) and in-hospital mortality (AUC:0.741, 95% CI: 0.715–0.767) predictive performance. Kaplan–Meier curve analysis reflected that those in the high-risk group showed lower survival compared to the low-risk group in both training and validation sets (all *P* < 0.01, Figures 5F, 6F and Supplementary Figure 3). Furthermore, DCA was also performed to determine the clinical utilities of the predictive risk model. The results indicated that the risk model was clinically useful (Figures 5E, 6E). Meanwhile, the novel risk model remains a firmly predictive tool for ICU mortality (OR: 1.96, 95% CI: 1.51–2.55; *P* < 0.05, Supplementary Figure 1) and in-hospital mortality (OR: 2.10, 95% CI: 1.71–2.58; *P* < 0.05, Supplementary Figure 1) across different subgroups. These results manifested that except for AKI, WBC-related biomarkers-based risk model could also markedly predict secondary clinical outcomes in critically ill patients with IS.

## Discussion

Herein, this study for the first time explored a novel risk model based on WBC-related biomarkers to predict AKI and other clinical outcomes in patients with IS admitted to the ICU. This retrospective study indicated that AKI was a common complication of IS admitted to the ICU. WLR, WBR, WHR, and NLR could independently predict AKI, and a novel risk model was established based on WLR, WBR, WHR, and NLR. This risk model exhibited good prediction performance both in AKI and other clinical outcomes (hemorrhage, persistent AKI, AKI progression, ICU mortality, and in-hospital mortality) in both training and validation sets.

Ischemic stroke is a major cause of death and disability worldwide. About 20% of patients with IS require care in an ICU (20). Although advances have been made to improve prognosis among critically ill patients with IS, many complications occurred during admittance to ICU including AKI. According to previous reports, the incidence of AKI following IS ranges from 0.82 to 26.68% (21). The AKI incidence in the ICU would be higher due to more comorbidities. One study investigated the epidemiology of AKI in 381 patients with stroke admitted to ICU and found that 115 (30.18%) patients developed AKI (22). Zhang et al. explored the incidence of AKI in critical care patients with acute cerebrovascular disease and found that 18.3% developed AKI (23). The incidence of AKI in the present study (28.4%) falls along the spectrum described in previous studies (22, 23). Patients with IS who developed AKI exhibited poor prognoses. A meta-analysis reported that patients with stroke who developed AKI increased the risk of mortality, degree of disability, length of hospital stay and hospitalization costs, and cardiovascular events (4). However, the precise mechanism underlying the high incidence of AKI in critically ill patients with IS remains largely unknown.

Previous studies reported that brain–kidney interaction may explain this mechanism. A review concluded that the central pathway of the brain–kidney interaction may be *via* the central autonomic network and sympathetic nervous system, and the peripheral signaling pathway may be regulated by inflammatory responses (24). The inflammatory response is tightly involved in the progression of a stroke, under stroke conditions, the body is in a pro-inflammatory state, and the C-reactive protein (CRP), IL-1β, IL-6, and tumor necrosis factor-α (TNF-α) were significantly increased following stroke (25, 26), and these inflammatory factors were all reported to be associated with the development of AKI (27, 28). Furthermore, integrated WBC-related inflammatory biomarkers such as NLR, PLR, and LMR have been reported to be involved in the progress of acute IS (11, 12, 29), and NLR, PLR, and LMR could also predict AKI in different settings (30–32). However, whether these WBC-related biomarkers could predict AKI in patients with IS admitted to ICU has been unexplored. Gao et al. previously reported that preoperative WBC to hemoglobin ratio (WHR), WBC to monocyte ratio (WMR), and platelet to lymphocyte ratio (PLR) associated with the prognosis of bladder cancer, and a risk model established based on WHR, WMR, and PLR showed good prognosis-predictive performance (33). In line with the previously reported (33), in this study, WLR, WBR, WHR, and NLR were independent prognostic predictors of AKI, and then WLR, WBR, WHR, and NLR were combined to construct a novel risk model.

Previous studies reported the different risk models to predict AKI in patients with IS. Zhu et al. developed a nomogram that includes blood urea nitrogen (BUN), creatinine, red blood cell distribution width (RDW), heart rate (HR), Oxford Acute Severity of Illness Score (OASIS), the history of congestive heart failure (CHF), the use of vancomycin, contrast agent, and mannitol and found that nomogram showed good predictive ability (34). For ICU patients, the SIRS, OASIS, APS III, SAPS II, and CCI scores are fundamental individualized risk assessments for characterizing disease severity and degree of organ dysfunction. This novel risk model in the present study showed better AKI prediction than those traditional score systems (OASIS, APS III, SAPS II, and SIRS) which are commonly used in the ICU. The SOFA score has been widely used to evaluate organ dysfunction in the ICU. Chang et al. used the SOFA score to predict ICU mortality in patients coexisting with AKI admitted to ICU and indicated that the SOFA score was a good prognosis-predictive tool (35), and this risk model also exhibited comparable AKI predictive ability to the SOFA score. Meanwhile, we also applied this model to predict other clinical outcomes, and the results demonstrated that this model could also predict hemorrhage, persistent AKI, AKI progression, ICU mortality, and in-hospital mortality in critically ill patients with IS.

This study has several strengths. First, to our knowledge, this is the first study to investigate the relationship between a WBC-related biomarkers-based risk model and AKI prediction in critically ill patients with IS. Second, we compared the novel risk model with the other scores system including SOFA score, OASIS score, APS III score, SAPS II score, SIRS score, Charlson index, and HAS-BLED score, and these scores systems were widely used in the ICU setting. Moreover, serum creatinine values were assessed during hospitalization and were used for these analyses. Finally, except for AKI prediction, this risk model was also used to predict other clinical outcomes including hemorrhage, persistent AKI, AKI progression, ICU mortality, and in-hospital mortality. However, some limitations should be addressed in this study. First, it is a retrospective study with data extracted from two public databases, and the results need to be verified by external validation. Second, the incidence of AKI with high heterogeneity according to different define diagnostic criteria. The incidence of AKI was 4.05% based on a coding definition for AKI, and 17.3% in a study utilizing creatinine-based definitions (4); this study used KDIGO-AKI criteria according to serum creatinine, and the incidence of AKI may be overestimated, and the AKI predictive ability of this risk model needed to be confirmed by further studies. Moreover, contrast-induced AKI is one of the common causes of AKI in clinical practice; however, due to the limitations of the MIMIC-IV and eICU-CRD databases, we were unable to obtain information about the contrast materials. Furthermore, stroke-specific characteristics such as NIHSS score, infarction area, and location are not included in the variables in this study due to the limitations of the MIMIC-IV database and the eICU-CRD database.

## Conclusion

We found that AKI appears to be a very common complication in patients with ischemic stroke admitted to the ICU. A novel risk model was established based on WBC-related biomarkers WLR, WBR, WHR, and NLR, which is easily accessible from the routine blood test, and this risk model depicted good prognosis-predictive performance including AKI and other clinical outcomes hemorrhage, persistent AKI, AKI progression, ICU mortality, and in-hospital mortality. This risk model may favor clinicians to take early interventions due to its simplicity. Further prospective studies are needed to determine the predictive performance of this study in the future.

## Data availability statement

The original contributions presented in this study are included in the article/Supplementary material, further inquiries can be directed to the corresponding authors.

## Ethics statement

The study was approved by the Institutional Review Board (IRB) of the Massachusetts Institute of Technology (MIT), and consent was obtained for the original data collection. Therefore, the ethical approval statement and the need for informed consent were waived for this manuscript.

## Author contributions

SL and ML conceived and designed the study. ML analyzed and interpreted the data and drafted the work. YY and YC participated in design of the study and assisted with revisions of the manuscript. WW and XZ extracted the data and take responsibility for the content of the manuscript including the data and analysis. All authors have approved the final version of the manuscript for submission and agreed to be accountable for all aspects of the work.
