# Supplementary material for: A novel risk model based on white blood cell-related biomarkers for acute kidney injury prediction in patients with ischemic stroke admitted to the intensive care unit
Source: Front Med (Lausanne). 2022 Dec 12;9:1043396. doi: 10.3389/fmed.2022.1043396 (PMC9790932; doi:10.3389/fmed.2022.1043396)
Supplement: Supplementary file 1 [file Data_Sheet_1.pdf]

Supplemental Table 1 Clinical outcomes analysis of high and low risk groups for patients in validation set

Outcomes	Low risk group	High risk group	Effect size	P value
N	1261	1114	-	-
Primary outcome				
AKI	320 (25.4)	468 (42.0)	0.358	<0.001
Secondary outcomes				
AKI severity[1]			0.364	<0.001
Stage I	84 (6.7)	125 (11.2)		
Stage II	172 (13.6)	228 (20.5)		
Stage III	64 (5.1)	115 (10.3)		
Persistent AKI[1]	107 (33.4)	210 (44.9)	0.236	0.002
AKI progression[1]	77 (24.1)	169 (36.1)	0.265	<0.001
Intracerebral hemorrhage	67 (5.3)	92 (8.3)	0.117	0.005
In-hospital mortality	201 (14.9)	172 (26.8)	0.189	<0.001
ICU mortality	132 (9.8)	102 (15.9)	0.132	0.002

AKI, acute kidney injury, ICU, intensive care unit.

[1]Excluded patients without the incidence of AKI.

Supplemental Table 2 Univariate and multivariate logistic regression analysis for clinical outcomes in validation set

Methods	OR (95%CI)	P value
For AKI		
Unadjusted	2.13 (1.79-2.54)	<0.001
Adjusted for model I	2.18 (1.83-2.61)	<0.001
Adjusted for model II	1.70 (1.40-2.06)	<0.001
Adjusted for model III	1.69 (1.39-2.05)	<0.001
For intracerebral hemorrhage		
Unadjusted	1.60 (1.16-2.23)	0.004

Adjusted for model I	1.59 (1.15-2.21)	0.006
Adjusted for model II	1.38 (1.08-1.95)	0.028
Adjusted for model III	1.34 (1.05-1.91)	0.035
For persistent AKI[1]		
Unadjusted	1.62 (1.21-2.18)	0.001
Adjusted for model I	1.62 (1.20-2.18)	0.002
Adjusted for model II	1.78 (1.31-2.44)	<0.001
Adjusted for model III	1.80 (1.31-2.48)	<0.001
For AKI progression[1]		
Unadjusted	1.78 (1.30-2.46)	<0.001
Adjusted for model I	1.82 (1.32-2.52)	<0.001
Adjusted for model II	1.69 (1.21-2.38)	0.002
Adjusted for model III	1.71 (1.21-2.42)	0.002

AKI, acute kidney injury, OR, odds ratio, 95%CI, 95% confidence index, Model I adjusted for age, gender, weight, ethnicity. Model II adjusted for model I plus comorbidities and Charlson comorbidity index, HAS-BLED score, score system, interventions, and drug usage. Model III adjusted for model II plus vital signs and laboratory results except for white blood count.

Supplemental Table 3 Univariate and multivariate COX regression analysis for clinical outcomes in validation set

Methods	HR (95%CI)	P value
For ICU mortality		
Unadjusted	2.49 (1.16-5.35)	0.020
Adjusted for model I	2.36 (1.09-7.74)	0.029
Adjusted for model II	2.55 (1.15-8.71)	0.025
Adjusted for model III	2.68 (1.19-6.02)	0.017
For in-hospital mortality		
Unadjusted	2.20 (1.38-3.51)	<0.001

Adjusted for model I	2.02 (1.26-3.23)	<0.001
Adjusted for model II	2.18 (1.15-4.16)	0.017
Adjusted for model III	2.02 (1.05-3.89)	0.035

AKI, acute kidney injury, HR, hazard ratio, 95%CI, 95% confidence index, Model I adjusted for age, gender, weight, ethnicity. Model II adjusted for model I plus comorbidities and Charlson comorbidity index, HAS-BLED score, score system, interventions, and drug usage. Model III adjusted for model II plus vital signs and laboratory results except for white blood count.

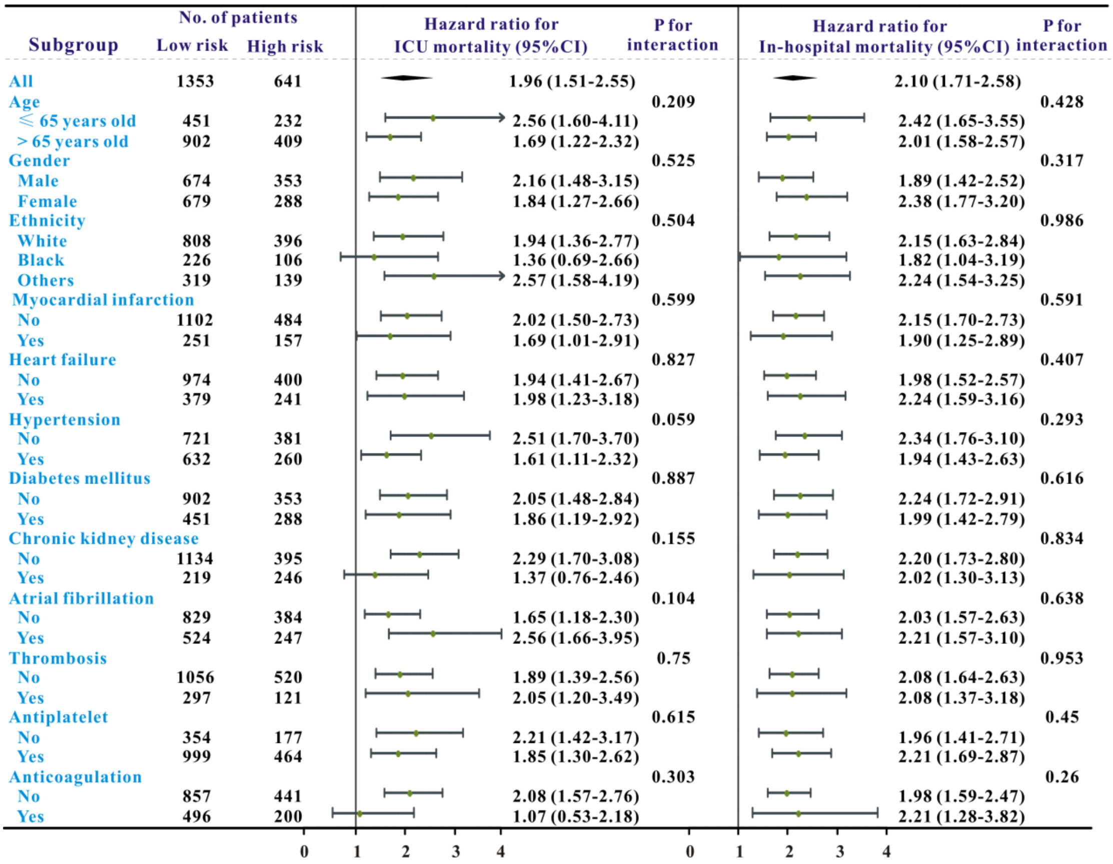

Supplemental Figure 1 The subgroup analysis of the risk score in individuals with IS for ICU mortality and in-hospital mortality in the validation set.

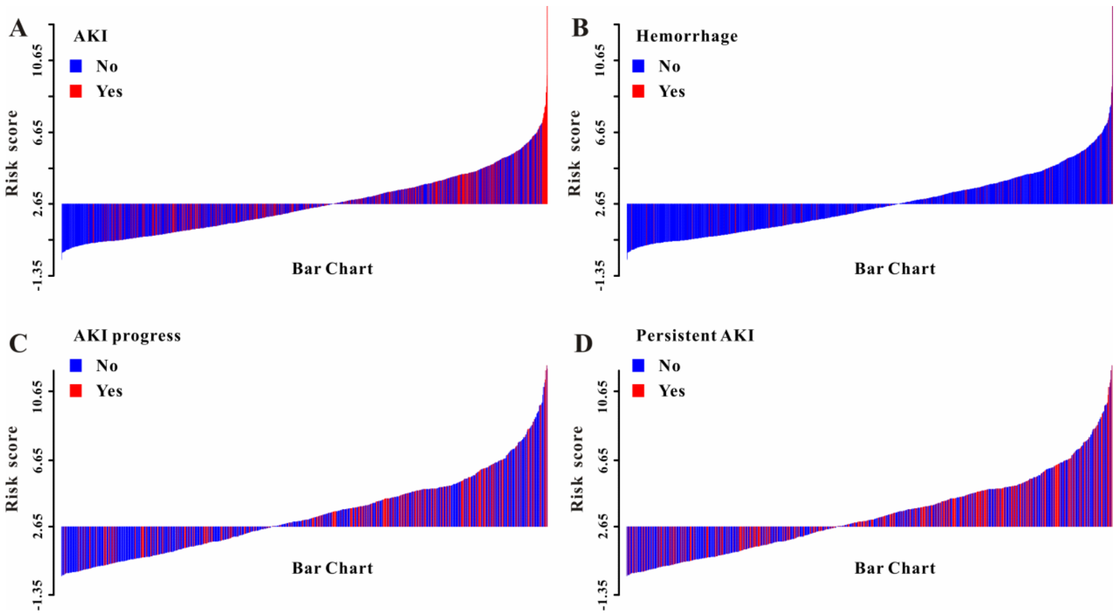

Supplemental Figure 2 The waterfall plots and forest plots of the high-risk group and low risk group for the prediction of AKI (**A**), intracerebral hemorrhage (**B**), AKI progression (**C**), and persistent acute kidney injury (**D**) for patients in the validation set.

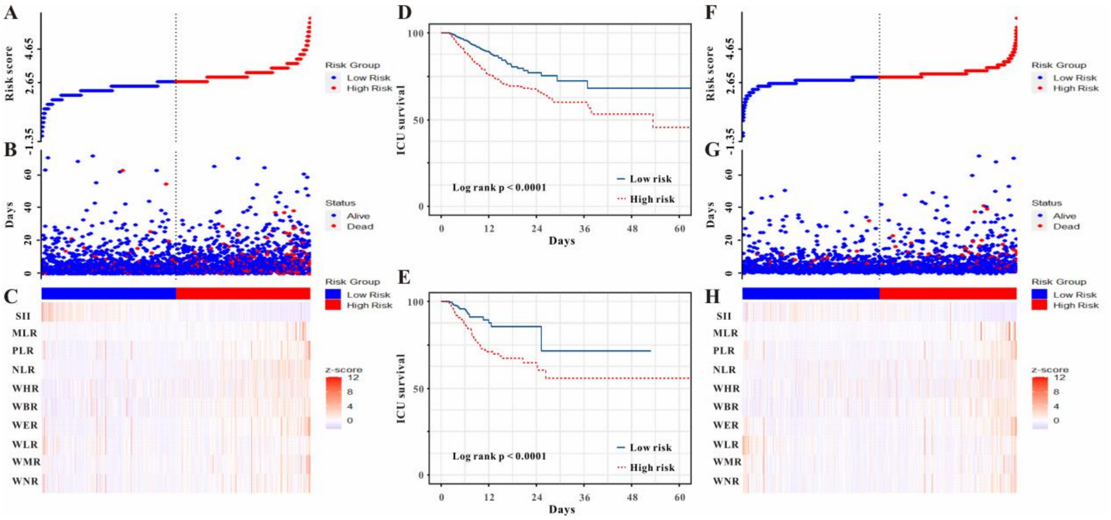

Supplemental Figure 3 The risk score was established to detect the in-hospital mortality of patients with IS in the validation set. All patients were distinguished into high and low risk based on the risk score (**A**), the relationship between survival time and prognosis of patients in the two corresponding groups (**B**), and the heatmap of inflammatory marks between the two groups (**C**). The Kaplan-Meier curves showing the in-hospital mortality (**D**) and ICU mortality of groups with different risk (**E**). The risk score was established to detect the ICU mortality of patients with IS in the validation set. All patients were distinguished into high and low risk based on the risk score (**F**), the relationship between survival time and prognosis of patients in the two corresponding groups (**G**), and the heatmap of inflammatory marks between the two groups (**H**).
